# Cyclophilin A/EMMPRIN Axis Is Involved in Pro-Fibrotic Processes Associated with Thoracic Aortic Aneurysm of Marfan Syndrome Patients

**DOI:** 10.3390/cells9010154

**Published:** 2020-01-08

**Authors:** Gianluca L. Perrucci, Erica Rurali, Maria Corlianò, Maria Balzo, Michela Piccoli, Donato Moschetta, Alessandro Pini, Raffaella Gaetano, Carlo Antona, Gustavo Egea, Gunter Fischer, Miroslav Malešević, Francesco Alamanni, Elisa Cogliati, Adolfo Paolin, Giulio Pompilio, Patrizia Nigro

**Affiliations:** 1 Unità di Medicina Rigenerativa e Biologia Vascolare, Centro Cardiologico Monzino-IRCCS, 20138 Milan, Italygiulio.pompilio@ccfm.it (G.P.);; 2 Centro Malattie Rare, Marfan Clinic, U.O. Cardiologia, ASST FBF-Sacco, 20157 Milan, Italy; 3 Centro di Cardiogenetica Vascolare, IRCCS Policlinico San Donato, San Donato Milanese, 20097 Milan, Italy; 4 Consiglio Nazionale delle Ricerche (CNR), Istituto di Ricerca e di Innovazione Biomedica (IRIB), 90146 Palermo, Italy; 5 Dipartimento di Scienze Biomediche e Cliniche “L. Sacco”, Università degli Studi, 20157 Milan, Italy; 6 Departament Biomedicina, Universitat de Barcelona and Institut d’Investigacions Mèdiques August Pi i Sunyer (IDIBAPS), 08036 Barcelona, Spain; 7 Max-Planck Institute for Biophysical Chemistry, 37077 Göttingen, Germany; 8 Enzymology department, Institute of Biochemistry and Biotechnology, Martin-Luther-University Halle-Wittenberg, 06120 Halle, Germany; 9 Unità Operativa di Chirurgia Cardiaca, Centro Cardiologico Monzino IRCCS, 20138 Milan, Italy; 10 Dipartimento di Scienze Cliniche e di Comunità, Università degli Studi, 20122 Milan, Italy; 11 Treviso Tissue Bank Foundation, 31100 Treviso, Italy; 12 Dipartimento di Chirurgia Cardiovascolare, Centro Cardiologico Monzino IRCCS, 20138 Milan, Italy

**Keywords:** thoracic aortic aneurysm, EMMPRIN, fibrosis, vascular smooth muscle cells, Cyclophilin A

## Abstract

Background: Marfan syndrome (MFS) is a genetic disease, characterized by thoracic aortic aneurysm (TAA), which treatment is to date purely surgical. Understanding of novel molecular targets is mandatory to unveil effective pharmacological approaches. Cyclophilin A (CyPA) and its receptor EMMPRIN are associated with several cardiovascular diseases, including abdominal aortic aneurysm. Here, we envisioned the contribution of CyPA/EMMPRIN axis in MFS-related TAA. Methods: We obtained thoracic aortic samples from healthy controls (HC) and MFS patients’ aortas and then isolated vascular smooth muscle cells (VSMC) from the aortic wall. Results: our findings revealed that MFS aortic tissue samples isolated from the dilated zone of aorta showed higher expression levels of EMMPRIN vs. MFS non-dilated aorta and HC. Interestingly, angiotensin II significantly stimulated CyPA secretion in MFS-derived VSMC (MFS-VSMC). CyPA treatment on MFS-VSMC led to increased levels of EMMPRIN and other MFS-associated pro-fibrotic mediators, such as TGF-β1 and collagen I. These molecules were downregulated by in vitro treatment with CyPA inhibitor MM284. Our results suggest that CyPA/EMMPRIN axis is involved in MFS-related TAA development, since EMMPRIN is upregulated in the dilated zone of MFS patients’ TAA and the inhibition of its ligand, CyPA, downregulated EMMPRIN and MFS-related markers in MFS-VSMC. Conclusions: these insights suggest both a novel detrimental role for CyPA/EMMPRIN axis and its inhibition as a potential therapeutic strategy for MFS-related TAA treatment.

## 1. Introduction

Marfan syndrome (MFS) is a connective tissue disease with an autosomal dominant inheritance and an estimated incidence of 1:5000 individuals [1]. This disorder is caused by mutations in the FBN1 gene encoding fibrillin-1, an extracellular matrix (ECM) protein that provides elasticity and structural support to several tissues [2]. At the molecular level, fibrillin-1 forms complex extracellular structures, called microfibrils, which modulate elastic fiber biogenesis and homeostasis and regulate the bioavailability/activity of different growth factors [3].

FBN1 mutations lead to impaired fibrillin-1 protein synthesis, secretion and/or incorporation in ECM, determining a microfibrillar architecture degeneration and destruction of the ECM integrity [4]. As a consequence, this genetic defect leads to pleiotropic manifestations, such as skeletal overgrowth, ocular lens luxation (i.e., ectopia lentis), and cardiovascular events as the progressive aortic-root dilation [5]. Overall, cardiovascular impairments dominate MFS patients’ prognosis. Indeed, the thoracic aortic dilatations worsening culminate in thoracic aortic aneurysms (TAA), which, in turn, often lead to the major cause of death in MFS patients as aortic dissection [1].

It is noteworthy that the therapy for MFS-associated TAA is, to date, purely surgical. Unfortunately, an effective pharmacological treatment for this life-threatening disease in MFS patients still remains unknown. Thus, the complete identification of the mechanisms contributing to MFS aneurysm formation and the subsequent identification of novel therapeutic targets are clinical imperatives for this patients’ category [6].

Over the past 25 years, novel insights were gained in the molecular biology of MFS, which have implicated the transforming growth factor-β (TGF-β)- and angiotensin II (AngII)-dependent pathways [7,8] as key culprits in TAA development. These findings suggest that other factors, in association with FBN1 mutations, may play an important role in TAA development and progression. However, a complete picture of the pathogenic mechanisms driving the onset and progression of aortic disease in MFS has not been achieved.

The receptor named extracellular matrix metalloproteinase (MMP) inducer (EMMPRIN) is strongly associated with several cardiovascular diseases [9]. Interestingly, EMMPRIN acts not only as an MMP activator, but also as a classical receptor regulating downstream pathways, specifically those involved in fibrosis and inflammation [10]. Of note, it was previously reported that EMMPRIN levels are strongly upregulated in VSMC of human non-syndromic TAA and its expression is induced in vitro by TGF-β and AngII [11,12,13]. Among EMMPRIN ligands important to be mentioned there are E-selectin, S100A9, and Cyclophilin A (CyPA) [14]. The latter is a peptidyl-prolyl cis–trans isomerase, object of several studies in the cardiovascular field [15,16]. Notably, we demonstrated that the deletion of CyPA in mice prevents the formation of abdominal aortic aneurysm (AAA) in response to AngII infusion [17]. Remarkably, we also showed that the expression of CyPA in VSMC, rather than in bone marrow–derived cells, is crucial in AAA development. To establish CyPA relevance in human aneurysmal disease, we discovered that AngII causes its release from VSMC derived from human AAA together with the activation of MMP-2. As EMMPRIN ligand, it was reported that CyPA/EMMPRIN binding activates ERK1/2 signaling pathway [18] and promotes also MMP cleavage. However, the contribution of CyPA/EMMPRIN axis in MFS-associated TAA has not been elucidated yet.

Here, we aimed to fill this gap, providing evidence about the role of CyPA/EMMPRIN axis as the molecular player in MFS and as a novel attractive therapeutic target. Specifically, we found that CyPA (i) is secreted from VSMC obtained by MFS patients’ TAA samples after treatment with AngII and (ii) orchestrates the activation of deleterious pathways leading to fibrosis in MFS through the upregulation and activation of EMMPRIN.

## 2. Materials and Methods

### 2.1. Data Availability

The data that support the findings of this study are available from the corresponding author upon reasonable request.

### 2.2. Patients Enrollment

Aortic tissue samples were collected in the surgery room from the dilated and non-dilated zone of MFS patients’ aortic aneurysm. From the same cohort of patients, and plasma samples were collected before the surgery procedure. The healthy control (HC) samples were provided by Treviso Tissue Bank Foundation. All MFS patients gave written informed consent for tissue collection and the procedure was approved by ASST FBF-Sacco Ethics Committee (Prot. N° 39138/2016, Milan, Italy). Aortic samples were stored in serum-free Medium 231 (Life Technologies, Carlsbad, CA, USA) and then divided into fragments dedicated to histology and molecular studies.

### 2.3. Immunostaining and Histological Assays

Aortic fragments assigned to histological studies were fixed in Carnoy’s solution, paraffin-embedded and 5-µm-thick sections were cut from each sample. To evaluate vascular elastic fiber integrity and collagen content in tissue, sections were stained with the Verhoeff-Van Gieson solution (Bio-Optica, Milan, Italy) following the manufacturer’s protocol. To perform immunohistological studies, sections were incubated with primary antibodies against CyPA (R&D system, Minneapolis, MN, USA) and EMMPRIN, also called CD147 (R&D System, Minneapolis, MN, USA), over-night (O/N) at 4 °C. As a negative control, species- and isotype-matched IgGs were incubated in place of primary antibodies. Slides were viewed with AxioScope microscope equipped with AxioCam camera (Carl Zeiss, Oberkochen, Germany). Densitometric analyses and elastic fiber length measurements were performed with AxioVision 4.7 software (Carl Zeiss, Oberkochen, Germany) by two blinded readers.

### 2.4. Aortic VSMC Isolation

Samples from aortic tunica media, stored in serum-free Medium 231 (Life Technologies, Carlsbad, CA, USA), were washed with Phosphate Buffer Saline (PBS), minced and digested O/N at 37 °C in a solution of 2 mg/mL collagenase type II (Worthington Biochemical Corporation, Lakewood, NJ, USA) in complete Medium 231, supplemented with Smooth Muscle Growth Supplement (SMGS, Life Technologies, Carlsbad, CA, USA). Then, the result of tissue digestion was filtered with 100 μm cell strainer, pelleted and plated in complete Medium 231. To improve cell growth, the day after isolation the medium was changed to remove the residual erythrocytes. Regarding MFS samples, cells used for the in vitro experiments were those isolated only by non-dilated zone.

### 2.5. ELISA Assays

For each patient enrolled in the study, 8 mL of peripheral whole blood was collected using EDTA Vacutainer tubes (Becton Dickinson, Franklin Lakes, NJ, USA). Plasma was obtained centrifuging the whole blood for 10 min at 2500× *g*, at 4 °C. CyPA serum levels of HC subjects and MFS patients were detected with an ELISA kit (USCN Life Science Inc., Wuham, China) following the manufacturer’s instructions.

TGF-β1 levels in VSMC conditioned medium after five days of 100 ng/mL CyPA and/or 250 ng/mL MM284 (CyPA inhibitor) treatment were detected with a sandwich Quantikine^®^ ELISA kit (R&D System, Minneapolis, MN, USA) following the manufacturer’s instructions.

### 2.6. MRNA Extraction and qRT-PCR Assay

After collection, aortic samples from healthy controls (HC) and Marfan syndrome patients (MFS) were stored at −80 °C in an appropriate amount of RNA Later solution (Sigma-Aldrich, Saint Louis, MO, USA). Once defrosted, aortic dried tissues were crushed by the Bessman Tissue Pulverizer (Spectrum Europe BV, Breda, Netherlands) and RNA was isolated with the TRIzol^®^ solution (ThermoFisher Scientific, Paislay, Scotland, UK). In parallel, after cell isolation, HC- and MFS-VSMC were treated with 100 ng/mL CyPA, 5 ng/mL TGF-β1, 1 μM AngII, 250 ng/mL MM284, 5 μg/mL EMMPRIN blocking antibody (Ancell, Bayport, MN, USA), or 5 μg/mL IgG isotype antibody (Ancell, Bayport, MN, USA). Cellular RNA was isolated by using a Total RNA Purification kit (Norgen Biotek corp., Thorold, Canada). Both for tissues and cells, the quantification of isolated RNA was determined with a spectrophotometer (ND-1000, NanoDrop^®^, EuroClone^®^). Reverse transcription was conducted with the SuperScript III (Invitrogen, Carlsbad, CA, USA) following the manufacturer’s instructions. qRT-PCR was performed with the use of the iQTM SYBR Green Super Mix (Bio-Rad Laboratories, Hercules, CA, USA). All reactions were performed in a 96-well format with the iQ5TM (Bio-Rad Laboratories, Hercules, CA, USA). The relative quantities of specific mRNA were obtained with the use of the comparative Ct method and were normalized to the Ribosomal Protein L32 (RPL32).

### 2.7. Western Blot and Slot Blot Analyses

Both aortic tissues and VSMC were lysed in cell lysis buffer (Cell Signaling Technology, Danvers, MA, USA) supplemented with protease and phosphatase inhibitor cocktails (Sigma-Aldrich, Saint Louis, MO, USA). Total protein extracts were subjected to SDS-PAGE and transferred onto a nitrocellulose membrane. The membranes were blocked for 1 h at room temperature in 5% non-fat dry milk in Wash Buffer (Tris Buffer Sulfate 1×, 0.1% Tween 20) and then incubated O/N at 4 °C with the appropriate primary antibody. For Slot blot analysis, 100 μL of VSMC conditioned media were directly loaded on nitrocellulose membrane with a pore size of 0.2 μm (Bio-Rad Laboratories, Hercules, CA, USA) by vacuum filtration. The membranes were blocked for 1 h at room temperature in 5% non-fat dry milk (Blotto, ChemCruz, Huissen, Netherlands) in Wash Buffer (Tris Buffer Sulfate 1×, 0.1% Tween 20) and then incubated O/N at 4 °C with the appropriate primary antibody. Primary antibodies used were specific for phospho-SMAD2/3 (Cell Signaling Technology, Danvers, MA, USA), SMAD2/3 (Cell Signaling Technology, Danvers, MA, USA), phospho-ERK1/2 (Cell Signaling Technology, Danvers, MA, USA), ERK1/2 (Cell Signaling Technology, Danvers, MA, USA), CyPA (R&D System, Minneapolis, MN, USA), EMMPRIN (R&D System, Minneapolis, MN, USA), AngII type 1 receptor (AT1R, AbCam, Cambridge, UK), and GAPDH (Santa Cruz Biotechnology, Dallas, TX, USA). The membranes were incubated with peroxidase-conjugated secondary antibodies (GE Healthcare, Chicago, IL, USA) for 1 h. Signals were visualized using the enhanced chemiluminescence Western blotting detection system (ThermoFisher Scientific, Paislay, Scotland, UK). Images were acquired with the Alliance Mini 2M (UVITec, Cambridge, UK), and densitometric analysis of membranes was performed using the Alliance Mini 4 16.07 software (UVITec, Cambridge, UK). Proteins were normalized according to the GAPDH signal.

### 2.8. ImageStreamX Imaging Flow Cytometry

For CyPA and EMMPRIN cellular localization experiments, VSMC were acquired by the ImageStreamX imaging cytometer (Amnis Corporation, Seattle, WA, USA), with 40× magnification and low flow rate/high sensitivity using the INSPIRE software. VSMC were treated with 100 ng/mL CyPA for 3 h, and then gently detached from Petri dishes by using the TrypLE™ Select solution (ThermoFisher Scientific, Paislay, Scotland, UK). Afterward, VSMC were pelleted, resuspended in FACS buffer (1× PBS, 0.1% Bovine Serum Albumin (BSA), and 5 mM EDTA) and incubated with conjugated primary antibodies for 10 min at room temperature. In the case of unconjugated primary antibody, cells were washed, resuspended in FACS buffer and incubated with a specific secondary antibody conjugated with a fluorescent dye. Data from 10,000 events per sample were collected and the percentage of the elements was calculated using the IDEA software (Amnis Corporation, Seattle, WA, USA). To assess the nuclear translocation of CyPA, primary antibody against CyPA (R&D system), secondary antibody FITC-conjuagated, and DRAQ5 nuclear dye (AbCam, Cambridge, UK) were used. In order to evaluate the membrane localization of EMMPRIN, a primary antibody against EMMPRIN-APC (TRA-1-85 Allophycocyanin MAb, Clone TRA-1-85, R&D System, Minneapolis, MN, USA) was used. As cell membrane control, primary antibody against human leukocyte antigen-ABC (HLA-ABC) conjugated with phycoerythrin (PE) fluorescent dye (BD Pharmigen, San Jose, CA, USA) was used.

### 2.9. Sircol Assay

Total soluble collagen content in cell lysates and supernatant from HC- and MFS-VSMC, treated for five days with 100 ng/mL CyPA and/or 250 ng/mL MM284, was measured using Sircol soluble collagen assays (Biocolor, Carrickfergus, UK) as described in the manufacturer’s protocol. The quantity of collagen was calculated according to the kit standard curve.

### 2.10. Statistical Analyses

Quantitative results are expressed as mean ± SD. Statistical significance was evaluated with GraphPad Prism 5. Variables were analyzed by two-way ANOVA with Bonferroni’s post-test or Student’s *t*-test, as appropriate. A value of *p* ≤ 0.05 was deemed statistically significant.

## 3. Results

### 3.1. MFS Patients’ Thoracic Aortic Aneurysm Shows Increased Fibrosis and Activation of TGF-β1 Signaling

**Figure 1:**
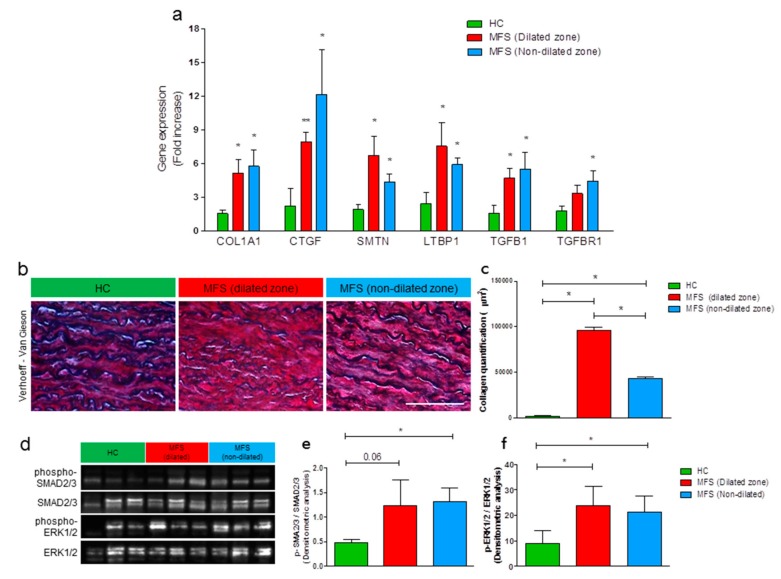
Collagen deposition, disease-related gene expression, and TGF-β-dependent pathways activation are higher in thoracic aortas of Marfan syndrome (MFS) patients than healthy controls. (**a**) Expression of MFS-related genes in total RNA extracts of thoracic aortas from healthy controls (HC) (green bars), MFS dilated (red bars), and MFS non-dilated zones (blue bars). qRT-PCR analyses have been performed in triplicate and data are shown as fold change ± SD, n = 5. Student’s *t*-test: * *p* < 0.05, ** *p* < 0.01. (**b**) Representative images of Verhoeff–Van Gieson staining on HC (left panel) and MFS patient aortas (dilated zone, central panel; non-dilated zone, right panel). Magnification = 20×. Scale bar = 200 μm. (**c**) Collagen quantification data are shown as mean ± SD, n = 5. Student’s *t*-test: * *p* < 0.05. (**d**) Western Blot of active phosphorylated form and total SMAD2/3 and ERK1/2 in total protein extracts of thoracic aortas from HC (green bars), MFS dilated (red bars), and MFS non-dilated zones (blue bars), and relative quantification. Data are shown as mean ± SD, n = 5. Student’s *t*-test: * *p* < 0.05. (**e**,**f**) Quantification of phospho-SMAD2/3 (**e**) and phospho-ERK1/2 (**f**) Western blot on thoracic aortic tissues. Data are shown as mean ± SD, n = 5. Student’s *t*-test: * *p* < 0.05.

To characterize TAA in MFS, we collected bioptic samples from dilated and non-dilated aorta of patients undergoing aortic replacement. MFS specimens were compared with thoracic aortic samples of HC. We evaluated the expression of principal genes involved in MFS disease by using total RNA extracts from the ascending aortic tissues. qRT-PCR analyses showed an upregulation of genes encoding several pro-fibrotic factors, such as collagen I (COL1A1) and connective tissue growth factor (CTGF) in aortic MFS patients’ samples vs. HC (Figure 1a). Similar results were obtained for SMTN, a gene encoding the typical VSMC marker smoothelin, and genes related to TGF-β1, such as TGFB1 itself, type 1 TGF-β receptor (TGFBR1), and latent TGF-β binding protein 1 (LTBP1).

To further investigate on the aortic wall structural integrity, we examined the elastic fiber disorganization/fragmentation and the collagen deposition, both typical features of MFS aortic tissue, by using the Verhoeff-Van Gieson staining. This specific staining highlights the former feature in black and the latter in pink/red (Figure 1b). As expected, we found an evident elastin fragmentation, evaluated and summarized in Table 1 as elastic fiber length, and a higher amount of collagen deposition in MFS samples when compared with HC (Figure 1c).

In order to assess the well-known over-activation of TGF-β1 pathways in MFS, we performed a Western blot analysis on total protein extracts of thoracic aortic samples isolated from HC and MFS patients (Figure 1d). As expected, we found a significant activation, in terms of higher expression levels of phosphorylated forms, of both transcription factors SMAD2/3 (Figure 1e) and ERK1/2 (Figure 1f) in MFS samples versus HC.

Altogether, these data confirm the pathologic events in patients’ samples and also support the key role of pro-fibrotic factors in the pathogenesis of MFS-associated TAA.

### 3.2. Expression Levels of EMMPRIN Are Higher in MFS vs. HC, both in vivo and in vitro

**Figure 2:**
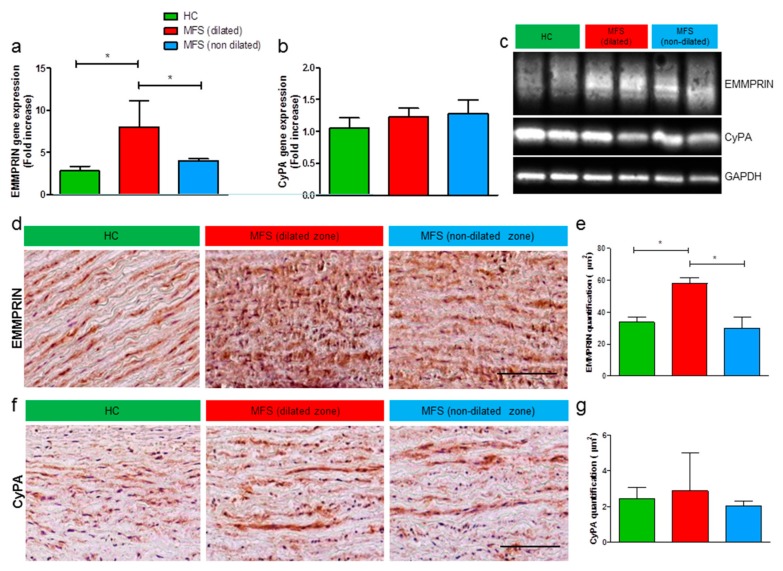
EMMPRIN expression levels are higher in the thoracic aorta of MFS patients. (**a**,**b**) EMMPRIN (**a**) and CyPA (**b**) gene expression in total RNA extracts of thoracic aortas from HC (green bars), MFS dilated (red bars) and MFS non-dilated zones (blue bars). qRT-PCR analyses were performed in triplicate and data are shown as fold change ± SD, n = 5. Student’s *t*-test: * *p* < 0.05. (**c**) Representative images of Western Blot analysis on EMMPRIN and CyPA in total protein extracts of thoracic aortas from HC (green bars), MFS dilated (red bars), and MFS non-dilated zones (blue bars). (**d**–**g**) Representative images of immunohistochemistry for EMMPRIN (**d**) and CyPA (**f**) on thoracic aorta of HC subjects (left panel) and MFS patients (dilated zone, central panel; non-dilated zone, right panel). Magnification = 20×. Scale bar = 200 μm. Quantification of immunohistochemistry for EMMPRIN (**e**) and CyPA (**g**) has been shown as mean ± SD, n = 5. Student’s *t*-test: * *p* < 0.05.

Based on a strong association between pathways/functions regulated by CyPA/EMMPRIN axis and major occurring events in MFS, we started our analysis of EMMPRIN and CyPA expression levels in MFS tissues compared to HC. Data described in Figure 2 provided evidence for an upregulation of EMMPRIN gene (Figure 2a) and protein (Figure 2c–e), specifically in the dilated zone of MFS aortic tissues when compared with HC samples and non-dilated zone of MFS aorta, both by Western blot (Figure 2c) and immunohistochemistry (Figure 2d,e) assays. Additionally, in an attempt to investigate the activator of this axis, we measured CyPA expression. Results revealed that CyPA expression is not modulated in MFS vs. HC aortic specimens, by qRT-PCR (Figure 2b), by Western blot (Figure 2c), and by immunohistochemistry (Figure 2f,g) analyses.

All these data suggest an important modulation of EMMPRIN but not of endogenous CyPA in MFS aneurysmal tissue, with particular relevance for the dilated zone of MFS-associated TAA.

Since VSMC have been described as pivotal players in aneurysm pathogenesis, these cells were explanted from the non-dilated zone of ascending aorta of MFS patients specimens (MFS-VSMC) and HC (HC-VSMC) and then characterized. Supplementary Figure S1 shows immunofluorescence characterization on HC- and MFS-VSMC, in which the VSMC identity was confirmed by the expression of typical smooth muscle cell markers, such as α-SMA and calponin. Moreover, in order to evaluate the expression of CyPA and EMMPRIN, multiple immunostainings were performed on VSMC. Specifically, EMMPRIN signal was detected together with α-SMA (Supplementary Figure S1a), while CyPA was evaluated together with calponin (Supplementary Figure S1b). Interestingly, this analysis showed a clearly visible difference in cell areas, greater in MFS-VSMC in comparison with HC-VSMC.

**Figure 3:**
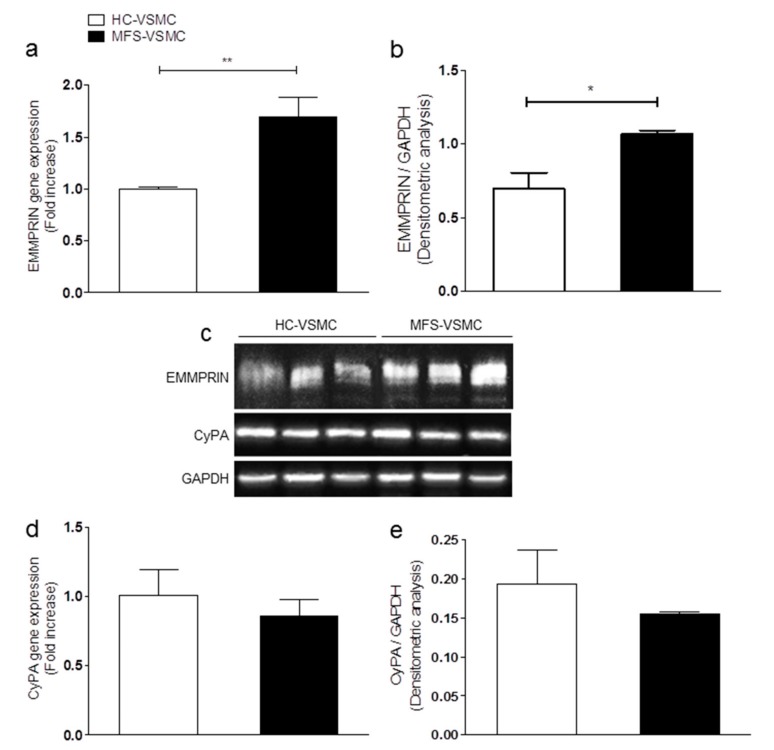
EMMPRIN expression levels are higher in vascular smooth muscle cells (VSMC) isolated from the thoracic aorta of MFS patients. (**a**,**d**) EMMPRIN (**a**) and CyPA (**d**) gene expression in RNA extracts of HC-VSMC (white bars) and MFS-VSMC (black bars). qRT-PCR analyses have been performed in triplicate and data are shown as fold change ± SD, n = 5. Student’s *t*-test: * *p* < 0.05, ** *p* < 0.01. (**c**) Western Blot of EMMPRIN and CyPA proteins in total extracts of HC-VSMC and MFS-VSMC, n = 3. (**b**,**e**) Quantification of EMMPRIN (**B**) and CyPA (**e**) Western blot. Data are shown as mean ± SD, n = 3. Student’s *t*-test: * *p* <0.05.

Then, qRT-PCR and Western blot analyses on VSMC ascribed the responsibility for EMMPRIN overexpression to these cells (Figure 3a–c). Similarly to tissue results, no statistical differences in CyPA expression levels (Figure 3c–e) were detected between HC- and MFS-VSMC.

### 3.3. MFS-VSMC Show A Higher Expression of AT1R, an Enhanced Secretion of CyPA, and MFS Patients Display Higher Plasmatic CyPA Levels

To date, it is well known that CyPA can localize into the nucleus, but also in the cytoplasmic compartment. Thus, we compared CyPA specific localization in HC- and MFS-VSMC by Amnis ImageStreamX. This approach revealed that CyPA localization is prevalently cytoplasmatic, with no statistical differences between HC- and MFS-VSMC (Figure S2).

**Figure 4:**
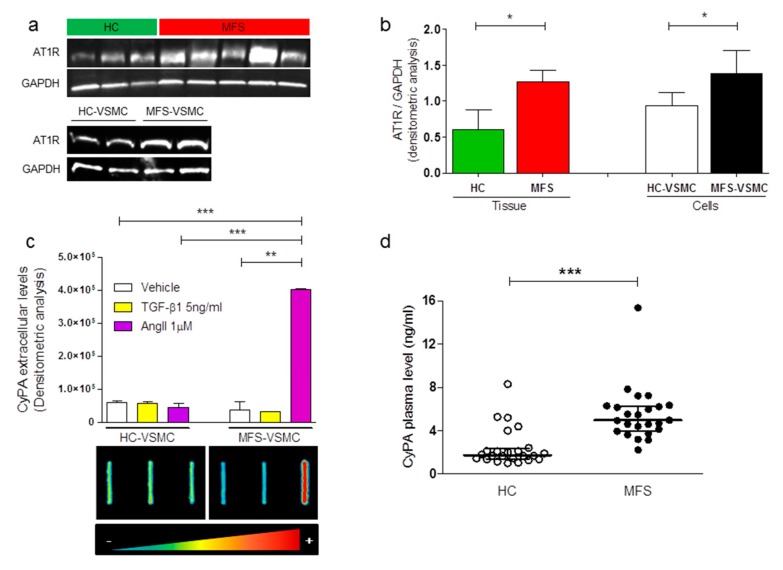
CyPA is secreted by MFS-VSMC after AngII treatment. (**a**) Western Blot of AT1R proteins in total protein extracts of thoracic aortas from HC (green bars, upper panel) and MFS dilated (red bars, upper panel), n = 8, and in total extracts of HC-VSMC and MFS-VSMC (lower panel), n = 4 for VSMC. (**b**) Quantification of AT1R Western blots. Data are shown as mean ± SD. Student’s *t*-test: * *p* < 0.05. (**c**) Slot-blot analysis and relative quantification of CyPA secreted in the conditioned media of HC- and MFS-VSMC, treated with vehicle (white bars), 5 ng/mL of TGF-β1 (yellow bars), and 1 μM AngII (purple bars), n = 5. Student’s *t*-test: * *p* < 0.05, ** *p* < 0.01, *** *p* < 0.0001. (**d**) ELISA assay on CyPA plasma levels of HC donors and MFS patients. Data are shown as median and IQR (InterQuartile Range 25–75%), n = 24/group. Mann-Whitney test: *** *p* <0.0001.

Although the initial evidence on the intracellular function of CyPA, recent studies revealed that this molecule can be secreted by several cell types in response to pathological stimuli [17,19,20]. In order to evaluate in our in vitro model whether CyPA is secreted after stimulation with specific MFS molecular mediators, we treated HC- and MFS-VSMC with TGF-β1 and AngII. The former is a well-known key player of TAA formation in MFS patients, the latter is an effective chemical stimulus involved in CyPA secretion by VSMC. Importantly, in 2013 it was reported that the AngII receptor type 1 (AT1R) is significantly upregulated in both aortic tissue and VSCM of MFS patients [21]. In order to provide a further quantitative result on AT1R protein expression levels, we performed a Western blot analysis for this AngII receptor on both aortic tissue and VSMC samples. As showed in Figure 4a,b, AT1R expression levels resulted higher both in MFS-derived samples of aortic tissue and VSMC. After that, our analysis by Slot blot assay on the VSCM conditioned medium showed, consistently with literature data, a massive increase of CyPA secretion, specifically in MFS-VSMC after AngII treatments (Figure 4c).

Several studies reported high circulating levels of CyPA in patients with numerous cardiovascular diseases [10,22,23,24,25]. Therefore, we next investigated whether circulating exogenous CyPA (CyPA) levels in MFS patients’ plasma may differ from those in HC, recapitulating the in vitro results. As reported in Figure 4d, MFS patients (n = 24) with thoracic aortic aneurysm showed a statistically significant increase in secreted levels of CyPA when compared with age- and sex-matched HC.

These data suggest a specific alteration of CyPA/EMMPRIN axis in MFS-VSMC. In fact, while CyPA is prevalently secreted into the bloodstream, its receptor EMMPRIN is locally enhanced in TAA of MFS patients.

### 3.4. CyPA Stimulates EMMPRIN Expression and Activation

**Figure 5:**
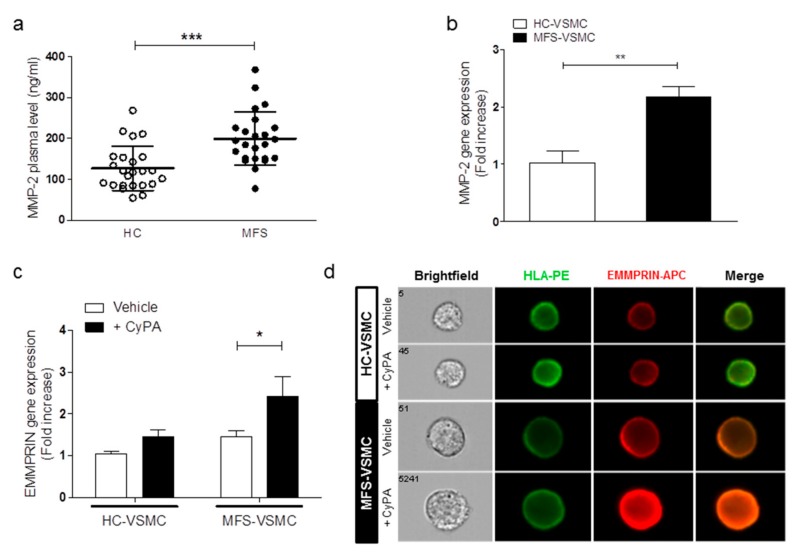
MMP-2 is up-regulated in MFS and exogenous CyPA promotes EMMPRIN gene and protein expression in MFS-VSMC. (**a**) ELISA assay on MMP-2 plasma levels of HC donors and MFS patients. Data are shown as mean ± SD, n = 24/group. Student’s *t*-test: *** *p* < 0.0001. (**b**) MMP-2 gene expression in RNA extracts of HC-VSMC (white bars) and MFS-VSMC (black bars). qRT-PCR analyses have been performed in triplicate and data are shown as fold change ± SD, n = 5. Student’s *t*-test: ** *p* < 0.01. (**c**) EMMPRIN gene expression levels in HC-VSMC and MFS-VSMC, after 100 ng/mL CyPA treatment. qRT-PCR analyses have been performed three times in triplicate and data are shown as fold change ± SD, n = 5. * *p* < 0.05 (**d**) Representative images of immunofluorescence on HC-VSMC and MFS-VSMC, after 100 ng/mL CyPA treatment, in brightfield, HLA-PE antibody, EMMPRIN-APC antibody, and merge. Cell images have been generated in real-time by Amnis imaging flow cytometer ImageStreamX with a magnification of 40×.

The most known EMMPRIN molecular function is that of matrix metalloprotease activator, among which the most important and active at the aneurysmal thoracic aortic tissue is MMP-2 [11,12,13]. In order to verify the MMP-2expression levels in the plasma of our cohort of patients as well as in the VSMC in vitro model, we performed an ELISA and a qRT-PCR assays, respectively. The results of both these assays confirmed that MMP-2 was significantly upregulated both in the plasma of MFS patients (Figure 5a) as well as gene expression in MFS-VSMC (Figure 5b).

Taking into account that CyPA is the ligand of EMMPRIN, that this binding is able in activating EMMPRIN, and that CyPA levels were higher in MFS patients’ plasma as well as in conditioned medium of MFS-VSMC after AngII stimulation, we next evaluated the direct effect of the exogeneous CyPA administration on MFS-VSMC. In order to assess whether CyPA treatment may influence cell proliferation or induce MFS-VSMC apoptosis, we performed two different FACS analyses. By using BrdU, we obtained no statistical differences in cycle phases of MFS-VSMC (Figure S3a), thus CyPA does not induce cell cycle transition in MFS-VSMC. Similarly, by using the Annexin V antibody, we demonstrated that CyPA does not induce apoptosis in MFS-VSMC (Figure S3b,c).

Results showed that CyPA (100 ng/mL) treatment led to a significant overexpression of EMMPRIN gene (Figure 5c) in MFS- vs. HC-VSMC. Concerning EMMPRIN protein levels, the ImageStreamX analysis confirmed the EMMPRIN localization on VSMC membranes and further highlighted the EMMPRIN overexpression after CyPA treatment, specifically on cell membranes of MFS-VSMC (Figure 5d).

To better understand whether EMMPRIN was functionally active in our setting, we took advantage of its capability to directly activate MMPs. Thus, HC- and MFS-VSMC were analyzed by an in situ zymography (Supplementary Methods) after treatments with CyPA and a selective antibody against the EMMPRIN extracellular domain. The immunofluorescence images showed a higher MMP activation after CyPA treatment, which is extrapolated by enhanced emission of a green-fluorescent signal by cleavages of engineered gelatin (Figure S4a). The quantification of FITC-positive areas revealed an overall higher activity of EMMPRIN in MFS- vs. HC-VSMC, and overall in MFS-VSMC after CyPA treatment (Figure S4b). Similarly, the CyPA/EMMPRIN axis inhibition mediated by MM284 led to significantly decreased levels of MMP-2 gene expression in MFS-VSMC (Figure S4c).

These data reveal that the treatment of MFS-VSMC with CyPA leads not only to a higher expression of EMMPRIN, but also to its increased activity.

### 3.5. Blocking CyPA/EMMPRIN Axis Reverts the MFS-Related Expression of Pro-Fibrotic Mediators

It is nowadays well established that MFS condition is strongly associated with the activation of TGF-β canonical (SMAD2/3) and non-canonical (ERK1/2) pathways which, in turn, lead to the upregulation of downstream genes. Moreover, several studies reported the capability of EMMPRIN as CyPA receptor in activating ERK1/2 signaling pathway [18,26,27].

Thus, we evaluated whether CyPA/EMMPRIN axis may participate in these molecular pathways by means of CyPA administration together with a novel compound as MM284. MM284 selectively block CyPA, is impermeable to cell membranes, and consequently, disables CyPA interaction with EMMPRIN [28].

To this aim, we treated MFS-VSMC with recombinant CyPA with/without MM284 to further investigate the modulation of MFS-related genes and signaling pathways.

**Figure 6:**
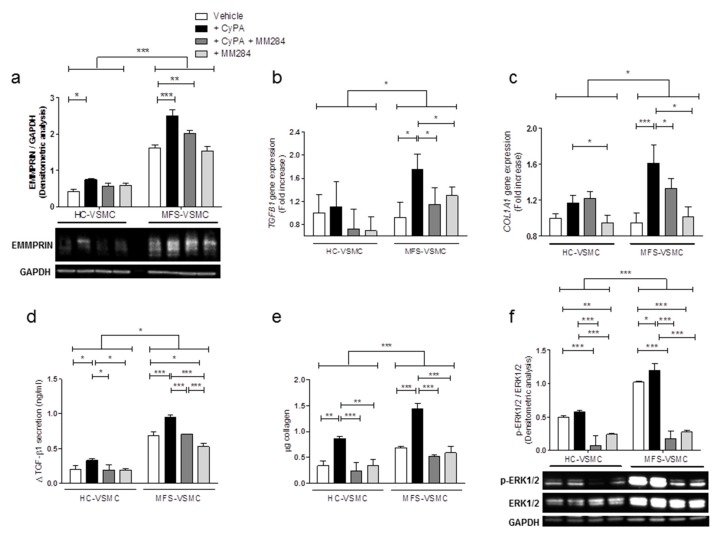
CyPA treatment activates ERK1/2 pathway in MFS-VSMC and promotes pro-fibrotic gene upregulation. (**a**) EMMPRIN expression levels in HC-VSMC and MFS-VSMC, after 100 ng/mL CyPA, 250ng/mL MM284, and treatment with both molecules. Protein quantification data are shown as mean ± SD, n = 5. GAPDH has been used as loading control. Two-way ANOVA and Bonferroni’s post-test: * *p* < 0.05, ** *p* < 0.01, *** *p* < 0.0001. (**b**,**c**) TGFB1 (**b**) and COL1A1 (**c**) gene expression in RNA extracts of HC-VSMC and MFS-VSMC, following treatment with 100 ng/mL CyPA, 250ng/mL of MM284, and CyPA+MM284. qRT-PCR analyses have been performed three times in triplicate and data are shown as fold change ± SD, n = 5. Two-way ANOVA and Bonferroni’s post-test: * *p* < 0.05, *** *p* < 0.0001. (**d**) ELISA assay for TGF-β1 in HC-VSMC and MFS-VSMC conditioned medium, after five days of treatment with 100 ng/mL CyPA, 250 ng/mL MM284, and CyPA + MM284. Analyses have been performed three times in triplicate and data are shown as mean ± SD, n = 5. Two-way ANOVA and Bonferroni’s post-test: * *p* < 0.05, *** *p* < 0.0001. (**e**) Quantification of total collagen levels in HC-VSMC and MFS-VSMC after five days of treatment with 100 ng/mL CyPA, 250 ng/mL MM284, and CyPA+MM284. Experiments have been performed three times in triplicate and data are shown as mean ± SD, n = 5. Two-way ANOVA and Bonferroni’s post-test: * *p* < 0.05, ** *p* < 0.01, *** *p* < 0.001. (**f**) Western Blot for active phospho-ERK1/2 and total ERK1/2 proteins in protein extracts of HC-VSMC and MFS-VSMC after 100 ng/mL CyPA, 250ng/mL MM284, and CyPA+MM284 treatment. Protein quantification data are shown as mean ± SD, n = 5. Total ERK1/2 has been used as loading control. Two-way ANOVA and Bonferroni’s post-test: * *p* < 0.05, ** *p* < 0.01, *** *p* < 0.0001.

Firstly, we confirmed by Western blot that the CyPA treatment significantly increased the EMMPRIN protein expression levels both in HC- and MFS-VSMC. Secondly, we observed that CyPA inhibition restored EMMPRIN levels to those of untreated conditions selectively in MFS-VSMC (Figure 6a). Thirdly, 100 ng/mL of CyPA treatment upregulates TGFB1 and COL1A1 gene expression levels exclusively in MFS-VSMC, while CyPA inhibitor limited these gene expression levels to the same values of untreated conditions (Figure 6b,c). These effects were further confirmed also in protein expression levels, since MM284 (250 ng/mL) notably limited CyPA-mediated effects on TGF-β1 and collagen protein expression (Figure 6d, e). At last, the same experimental conditions were adopted to detect the in vitro activation of ERK1/2 signaling. Also, in this case, MFS-VSMC showed an overall upregulation of this pro-fibrotic pathway in comparison with HC-VSMC, the CyPA treatment further enhanced phospho-ERK1/2 expression levels, while MM284 (250 ng/mL) mitigated CyPA effects in this signaling pathway stimulation (Figure 6f).

Taking all these data together, we demonstrated the critical role played by CyPA/EMMPRIN axis in the modulation of MFS-related key pathway activation and gene expression, providing a proof-of-concept that the blockage of this axis may offer a novel therapeutic target to treat TAA in MFS patients.

## 4. Discussion

This study addressed, for the first time, a strong involvement of CyPA/EMMPRIN axis in the modulation of detrimental processes, strictly connected to fibrosis, leading to TAA development of patients with MFS disease. Specifically, we discovered that the in vitro treatment of VSMC isolated by MFS patients’ aortas with exogenously administered CyPA leads to: (i) the overexpression and activation of the CyPA receptor EMMPRIN, (ii) the over-activation of ERK1/2 signaling pathway, and (iii) the subsequent upregulation of typical pro-fibrotic mediators, such as TGF-β1 and collagen I. Altogether, these data suggest a deep involvement of CyPA/EMMPRIN axis is crucial and detrimental processes involved in MFS-associated TAA.

Primum movens of our hypothesis started from the analysis of massive collagen deposition in the dilated zone of MFS patients’ thoracic aorta after surgery. This initial observation suggested a larger progression of the fibrotic process in these subjects and was supported by previous insights on the pro-fibrotic histological changes of the aneurysmal aorta [29]. Over time, several studies discovered that TGF-β and AngII actively participate as causative players in the aortic aneurysmal development [30]. More specifically, fibrillin-1 alterations actively contribute to the TGF-β massive release from its latent form stored in thoracic aortic ECM [31]. Our histological data was further confirmed at a molecular level by the overexpression of disease-related genes and the activation of specific pro-fibrotic signaling pathways.

**Figure 7:**
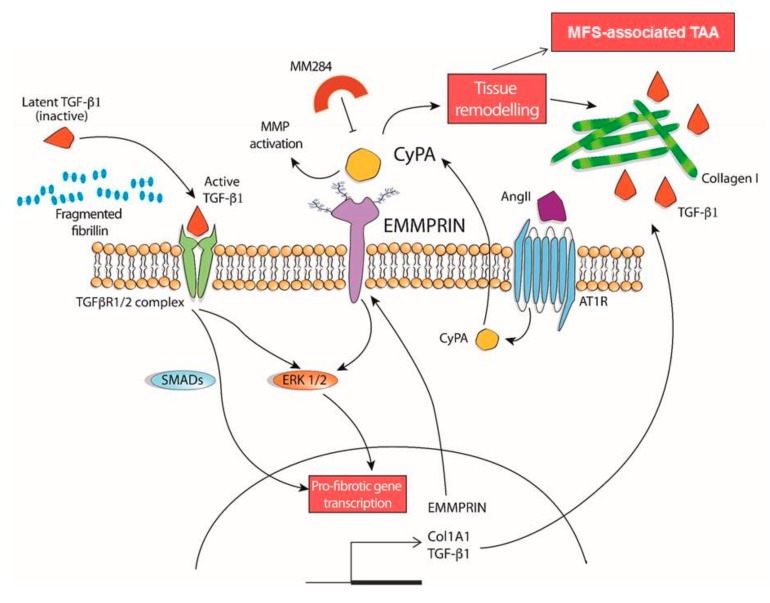
Proposed mechanism of CyPA/EMMPRIN axis involvement in TAA associated with MFS.The ECM of thoracic aorta in MFS patients is characterized by fragmented fibrillin-1, which determines TGF-β1 release. Active TGF-β1 binds its receptor and stimulates both the SMAD2/3 (canonical) and ERK1/2 (non-canonical) pathways, leading to the transcription and translation of pro-fibrotic factors, such as collagen and TGF-β1 itself. AngII stimulus leads to the secretion of CyPA, which binds its receptor EMMPRIN. The CyPA/EMMPRIN axis triggers (i) ERK1/2 pathway (non-canonical signaling, shared with TGF-β1), (ii) EMMPRIN, which, in turn, induces MMP activation, and (iii) the expression of typical downstream ERK1/2 pro-fibrotic mediators, involved in tissue remodeling of MFS-associated TAA. The inhibitor MM284 reverses all the deleterious effects of CyPA in MFS-VSMC.

On the basis of this strong rationale offered by these remarks, we wanted to in-depth investigate the involvement of CyPA/EMMPRIN axis in the MFS aortic pathological context. In fact, an important involvement of both CyPA and EMMPRIN in several cardiovascular diseases and also in tissue remodeling has been already reported by literature [9]. More specifically, CyPA was reported to be critically involved in vascular remodeling [32], cardiac hypertrophy [33], as well as in AAA formation [17], where its release by VSMC negatively contributes in disease development. Concerning EMMPRIN, this molecule was object of study in atherosclerosis [34,35], cardiac fibrosis in a pressure overload murine model [36], scar tissue formation after myocardial infarction [37], inflammatory cardiomyopathy [10], and tissue remodeling [12,13], revealing its major activity in MMP and pro-fibrotic signaling activation. Importantly, among all these cardiovascular pathologies, Chen et al. reported an AngII-dependent increase in EMMPRIN expression levels on VSMC, in non-syndromic form of both TAA and AAA [11]. Interestingly, our results on EMMPRIN gene and protein expression levels confirmed an upregulation in MFS aortic tissues, particularly in the dilated zone of aortic aneurysm, which is at the same time the most fibrotic aortic region in these patients. Corroborating these results, we recently observed diminished plasmatic levels of the soluble form of EMMPRIN in MFS patients, confirming the detrimental role played by EMMPRIN when its presence is localized on VSMC of aortic tissue [38]. On the other hand, the CyPA gene and protein levels did not reveal any significant difference in comparison with healthy controls. These counter-intuitive results might be explained by a different role/fate of CyPA in this pathological scenario. Different studies on intracellular CyPA reported a cytosolic localization of this molecule, but it is also well known that in the AAA context CyPA is secreted by VSMC after AngII stimulation [17]. Furthermore, this archetypal cell type of aortic wall is the foremost cellular element involved in MFS pathogenesis [39]. Noteworthy, unlike other adult cells, VSMC maintain notable plasticity [40], together with a peculiar ability to alternatively have a contractile or a synthetic phenotype. The latter renders VSMC more proliferative and also source of production of detrimental pro-fibrotic factors, as shown in Figure 7 [40,41,42]. This feature may be responsible for a larger area of MFS-VSMC in comparison with HC-VSMC that we observed in our experiments. A previous observation of AngII effects on VSMC together with the higher levels of AT1R in MFS samples led us to test this treatment also in our in vitro model of MFS-derived VSMC, in which we appreciated a massive secretion of CyPA specifically in conditioned medium of VSMC obtained from MFS thoracic aortas. This result met an astonishing analogy with the data concerning CyPA concentration in plasma samples of our MFS patients, confirming that the in vitro suggestions may have a pathophysiological counterpart in vivo.

Consistently, we found a strong upregulation of EMMPRIN gene and protein levels after CyPA treatment, corroborating the promoting activity of CyPA on the expression levels of its receptor in our patient-derived VSMC model. Previous studies reported that CyPA/EMMPRIN axis positively modulates ERK1/2 signaling pathway [43,44,45,46]. Our results confirm both the upregulation of ERK1/2 pathway after CyPA treatment and the subsequent overexpression of downstream pro-fibrotic mediators. Altogether, these results led us to hypothesize the involvement of the CyPA/EMMPRIN axis activation in the tissue remodeling of thoracic aorta in MFS patients. While the TGF-β secretion and collagen deposition are important hallmarks of fibrosis, MMPs play a crucial role in tissue remodeling. In fact, a determinant event in the pro-fibrotic ECM remodeling is represented by the activation of MMPs; more specifically, Segura et al. showed a strong overexpression of MMP-2 and MMP-9 in the aortic tunica media of MFS patients [47]. Moreover from further literature emerges the fundamental role of EMMPRIN in MMP-2 and -9 activation in different tissues and pathologies [12,13], including AAA [11]. The results of our work suggested the participation of EMMPRIN in MFS disease, not only as a receptor activating the ERK1/2 pro-fibrotic signaling pathway but also as an inducer of the MMP-2 activation.

In addition, it has been reported that the CyPA binding to EMMPRIN is necessary to enable it as an MMP inducer and the results collected demonstrate the proper functioning of this axis [48]. It is also worth it to point out that the fibrotic process can be considered as a double edge sword in cardiovascular pathophysiology, being, at the same time, an adaptive and protective mechanism, but also an irreversible remodeling process impairing organ function and architecture [49]. Therefore, fibrosis into the aortic wall should not be considered a pathological event, unless it becomes detrimental when passing an unknown threshold, who’s definition is not within the scope of this work. Nonetheless, unraveling a new pro-fibrotic central axis in MFS TAA may add new information for future investigations.

These results open a new perspective for TAA pharmacological research, which avoids direct TGF-β1 targeting. In the last years, in fact, published studies have generated conflicting results when targeting TGF-β1 itself and/or its downstream pathways for TAA treatment [7,8,50,51,52]. Of note, our experiments, taking advantage of in vitro blockade of the CyPA/EMMPRIN axis by MM284, further highlighted a strong involvement of this axis in TAA of MFS by reverting pro-fibrotic pathways including the secretion of the active form of TGF-β1. These results hint at a possible exploitation of cyclosporine analogs in counteracting TAA development in MFS. In this regard, additional in vivo experiments are needed to confirm this hypothesis. Several studies already provided evidence on MM284 beneficial role in cardiovascular conditions, such as myocarditis [53] and thrombi formation [54].

## 5. Conclusions

In conclusion, our results, summarized in Figure 7, demonstrate that AngII-related CyPA secretion can biochemically and functionally activate EMMPRIN in the dilated portion of MFS patients’ aorta by promoting the production of pro-fibrotic mediators and by simultaneously activating MMP. Since an etiological treatment for TAA is still lacking [39], this work has the potential to pave the way to new druggable pathways to be exploited for pharmacological TAA treatment development.

## Figures and Tables

**Table 1:** Elastic fiber length measurement in thoracic aortas of healthy controls (HC) and MFS patients.

	Elastic Fiber Length Mean (μm ± SD)	*p*
HC	235.94 ± 64.78	/
MFS (dilated zone)	116.07 ± 54.64	*
MFS (non-dilated zone)	97.93 ± 37.68	*

HC = healthy controls; MFS = Marfan syndrome. * = Student’s *t*-test *p*-value < 0.05 (vs. HC).

## Supplementary Materials

The following are available online at https://www.mdpi.com/2073-4409/9/1/154/s1, Figure S1: Cells isolated from aortic tunica media are VSMC and express CyPA and EMMPRIN; https://www.mdpi.com/2073-4409/9/1/154/s2, Figure S2: CyPA is localized into the cytoplasm of VSMC; https://www.mdpi.com/2073-4409/9/1/154/s3, Figure S3: CyPA does not alter cell proliferation and does not induce apoptosis in MFS-VSMC; https://www.mdpi.com/2073-4409/9/1/154/s4, Figure S4: CyPA/EMMPRIN axis inhibition decreases MMP expression and activity in MFS-VSMC.
