# Supplementary material for: Cyclophilin A/EMMPRIN Axis Is Involved in Pro-Fibrotic Processes Associated with Thoracic Aortic Aneurysm of Marfan Syndrome Patients
Source: Cells. 2020 Jan 8;9(1):154. doi: 10.3390/cells9010154 (PMC7016677; doi:10.3390/cells9010154)
Supplement: Supplementary file 1 [file cells-09-00154-s001.pdf]

Cyclophilin A/EMMPRIN axis is involved in pro-fibrotic processes associated with thoracic aortic aneurysm of Marfan syndrome patients

Gianluca L. Perrucci[1,*], Erica Rurali[1], Maria Corlianò[1], Maria Balzo[1], Michela Piccoli[1], Donato Moschetta[1], Alessandro Pini[2,3], Raffaella Gaetano[4], Carlo Antona[5], Gustavo Egea[6], Gunter Fischer[7], Miroslav Malešević[8], Francesco Alamanni[9,10], Elisa Cogliati[11], Adolfo Paolin[11], Giulio Pompilio[1,10,12°], Patrizia Nigro[1°]

Correspondence to: gianluca.perrucci@ccfm.it

Supplementary methods

Immunofluorescence on cells.

After growth medium removal, MFS-VSMC were fixed with 4% PFA, blocked with PBS supplemented with 5% BSA and incubated with primary antibody mixture against EMMPRIN (R&D System), CyPA (R&D System), α-SMA (Millipore), and calponin (Sigma) O/N at 4°C. As a negative control, species- and isotype-matched IgGs were incubated in place of primary antibodies. The secondary antibodies used were donkey anti-goat-Alexafluor 488(Life technologies), goat anti-mouse-AlexaFluor 594 (Life technologies for CyPA). Nuclei were stained with Hoechts 33342 (Life Technologies). Slides were viewed with Apotome.2 microscope equipped with AxioCam camera (Carl Zeiss).

Cell cycle and apoptosis assays.

The effects of CyPA and its inhibitor MM284 treatment on MFS-VSMC cell cycle and apoptosis were evaluated for 48 h in complete Medium 231 (Life Technologies). The cell cycle distribution after cell starvation was evaluated at Gallios flow cytometer (Beckman Coulter) by bromodeoxyuridine (BrdU) assay. Briefly, cells were treated with BrdU (Sigma) solution at a final concentration of 30 μM for 1 h in a CO2 incubator at 37 °C. Then, cells were washed in PBS, resuspended in PBS, fixed with 95% ethanol, and stored O/N at 4 °C. The day after, cells were pelleted, treated with 2 N HCl (Sigma), 0.2% Triton X-100 (Sigma) for 30 min at room temperature and then resuspended with 0.1 M Na2B4O7 (Sigma), pH 8.5 to neutralize acid. MFS-VSMC were washed in PBS, 0.1% BSA, 0.5% Tween 20 (Sigma), and incubated with antibody anti-BrdU-FITC (BD Bioscience) for 30 min at room temperature. Data from 20,000 events per sample were collected and the relative percentages of the cells in G0/G1, S, and G2/M phases of the cell cycle were calculated using Kaluza software (Beckman Coulter). For cell death experiment, MFS-VSMC were resuspended in FACS buffer (0.1% Bovine Serum Albumin, BSA and 5 mM EDTA in PBS) after treatment and incubated with AnnexinV-FITC antibody (eBioscience) for 10 min at room temperature. Then, cells were washed and resuspended in FACS buffer. Data from 10,000 events per sample were collected and the percentage of the elements was calculated using Kaluza software (Beckman Coulter).

In situ zymography.

VSMC were plated on chamber slides and, after 100 ng/mL CyPA and/or antibody against EMMPRIN treatments, incubated with the DQ gelatin (EnzChek® Gelatinase/Collagenase Assay Kit, Molecular Probe) following the manufacturer's instructions. After, cells were fixed with 4% paraformaldehyde (PFA) and incubated with the Hoechst solution (Thermo Fisher Scientific). Analyses were performed using a Zeiss LSM 710 confocal microscope. The Fluorescein isothiocyanate (FITC) signal related to cleaved gelatin was analyzed by ZEN 2008 software (Carl Zeiss) and normalized to the nuclei number. Counts were performed by two readers blinded to the treatment.

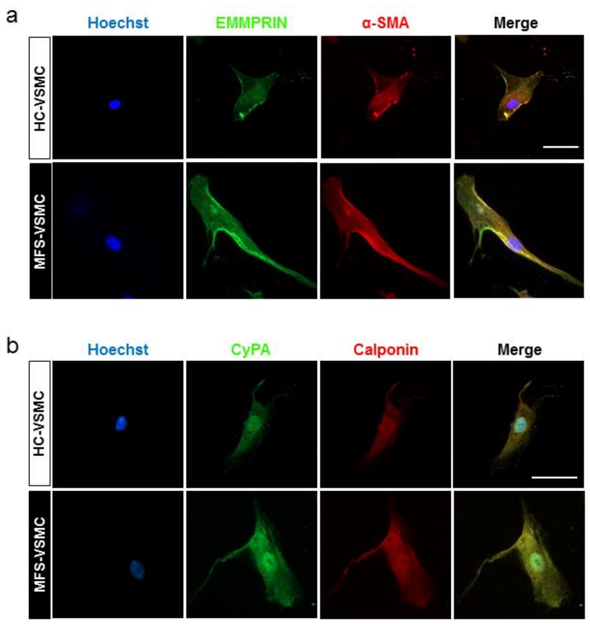

Figure S1 – Cells isolated from aortic tunica media are VSMC and express CyPA and EMMPRIN. (**a, b**) Representative images of immunofluorescence on HC-VSMC and MFS-VSMC isolated from thoracic aortas of HC subjects and from non-dilated zone of thoracic aortas of MFS patients. (**a**) Immunofluorescence images: Hoechst for nuclear staining (blue signal), EMMPRIN-AlexaFluor 488, α-SMA-AlexaFluo 594, and merge. Magnification=20X. Scale bar=50µm. (**b**) Immunofluorescence images: Hoechst for nuclear staining (blue signal), CyPA-AlexaFluor 488, calponin-AlexaFluo 594, and merge. Magnification=20X. Scale bar=50µm.

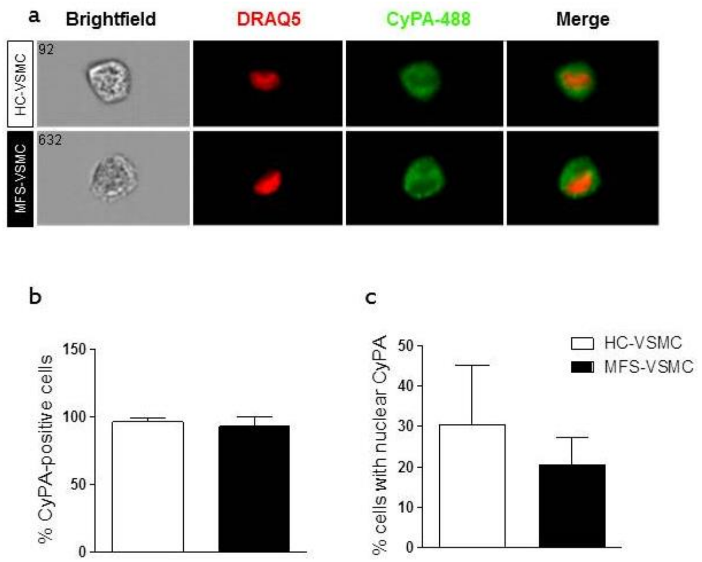

Figure S2 – CyPA is localized into the cytoplasm of VSMC.
(**a**) Representative images of immunofluorescence on HC-VSMC (upper lane) and MFS-VSMC (lower lane) isolated from thoracic aortas and MFS patients in brightfield, DRAQ5 nuclear staining, CyPA-AlexaFluor 488, and merge. Cell pictures are generated in real time by Amnis imaging flow cytometer ImageStream X with a magnification of 40X. (**b**) Fluorescence intensity bar graph reporting the percentage of CyPA-positive HC-VSMC and MFS-VSMC. 10,000 events have been acquired for each cell types. Results are shown as percentage ± SD. Student's t-test has been performed. (**c**) Bar graph reporting the percentage of HC-VSMC and MFS-VSMC positive for nuclear signal of CyPA. 10,000 events have been acquired for each cell types. Results are percentage ± SD. Student's t-test has been performed.

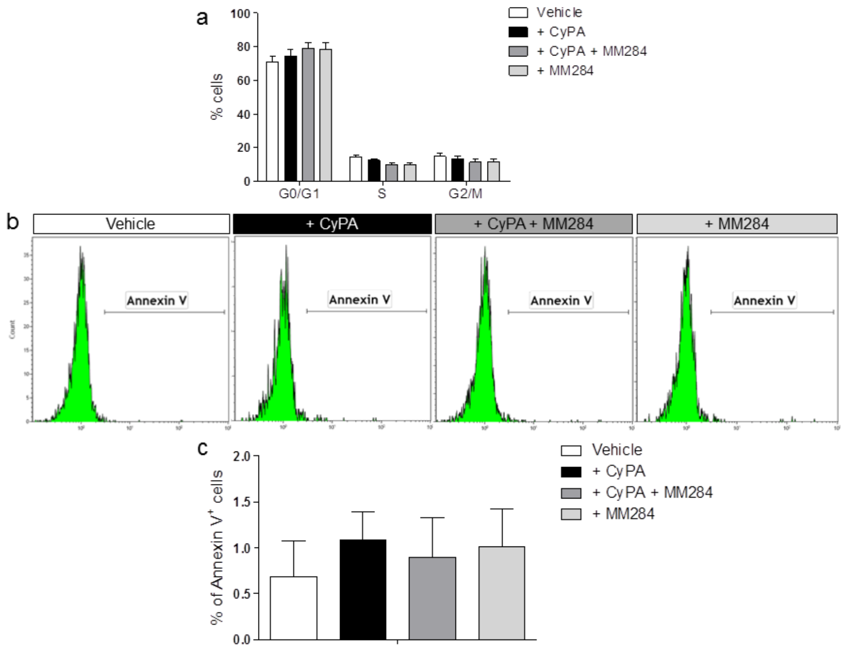

Figure S3 – CyPA does not alter cell proliferation and does not induce apoptosis in MFS-VSMC.
(**a**) FACS analysis of BrdU assay was performed on MFS-VSMC after 100 ng/ml CyPA, 250ng/ml MM284, and treatment with both molecules. Two-way ANOVA and Bonferroni's post-test were performed. (**b, c**) FACS analysis (**b**) and relative quantification (**c**) reporting the percentage of Annexin V-positive MFS-VSMC after 100 ng/ml CyPA, 250ng/ml MM284, and treatment with both molecules for 48 h. One-way ANOVA were performed.

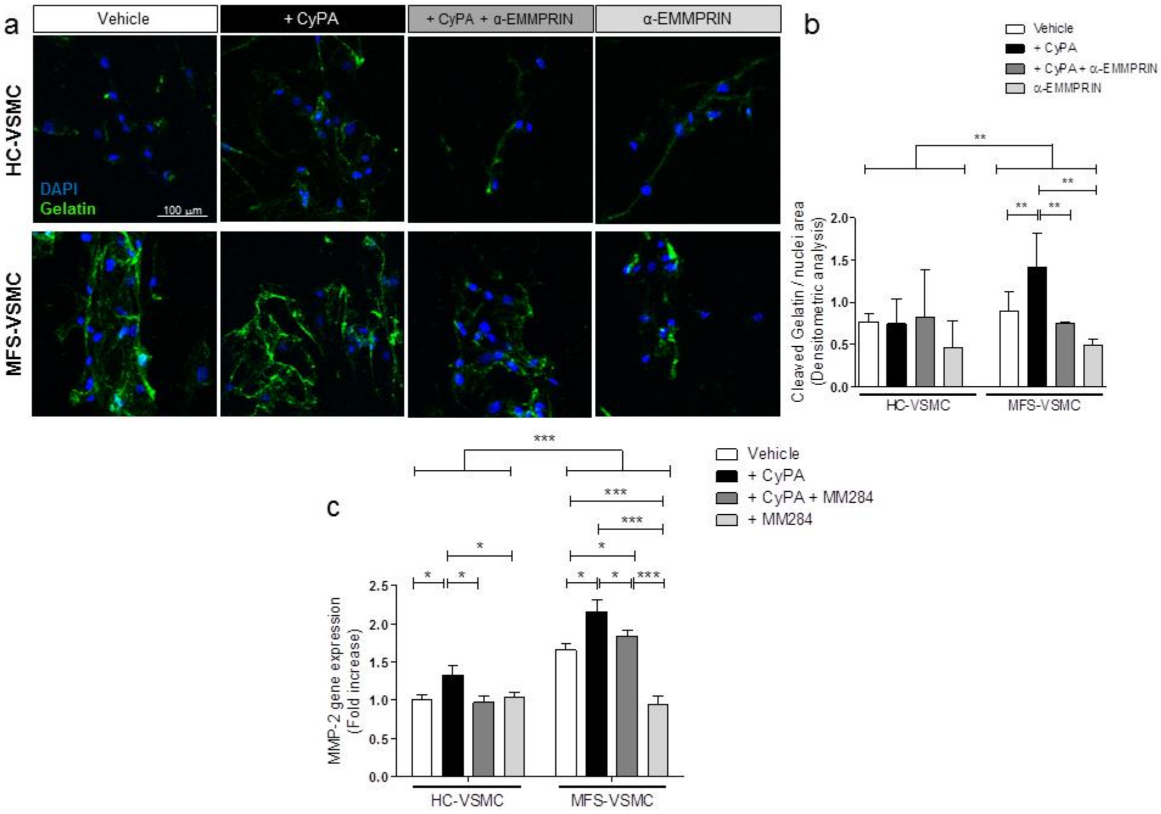

Figure S4 – CyPA/EMMPRIN axis inhibition decreases MMP expression and activity in MFS-VSMC.
(**a**) Representative images of in situ zymography immunofluorescence on HC-VSMC (upper lane) and MFS-VSMC (lower lane) after 5 days of treatment with 100 ng/ml CyPA, 5 µg/ml α-EMMPRIN antibody,

CyPA+α-EMMPRIN treatments. The pictures show DAPI nuclear staining (in blue) and cleaved gelatin-FITC (in green). Magnification=40X. Scale bar=100µm. (**b**) Quantification data of cleaved gelatin (green signal) normalized for nuclei area. Data are shown as mean ± SD, n=5. Two-way ANOVA and Bonferroni's post-test: $**p<0.01$. (**c**) MMP-2 gene expression in RNA extracts of HC-VSMC and MFS-VSMC, following treatment with 100 ng/ml CyPA, 250ng/ml of MM284, and CyPA+MM284. qRT-PCR analyses have been performed three times in triplicate and data are shown as fold change ± SD, n=5. Two-way ANOVA and Bonferroni's post-test: $*p<0.05$, $*p<0.05$; $***p<0.0001$.
